# Scientific Massage *versus* Ineffectual Rubbing

**Published:** 1921-09-24

**Authors:** F. Chaplin Hall

**Affiliations:** Secretary. 224-6-8 Great Portland Street, London, W. 1


### THE EDITOR'S LETTER-BOX.

**Correspondence on all subjects is invited, but we cannot in any way be responsible for the opinions expressed.**

### Scientific Massage versus Ineffectual Rubbing.

*To the Editor of* The Hospital.

Sir,—We are constantly receiving complaints from persons suffering from ailments, in the treatment of which scientific massage is supremely important, to the effect that the treatment received consists merely in what may be termed ineffectual rubbing. As Secretary of the Association of Certificated Blind Masseurs, and in the interests of its members, who are fully trained and qualified, the majority of them being soldiers who were blinded in the war, I shall be very grateful if you can find space in the columns of your valuable paper to state that skilled massage, as dissociated from mere rubbing, is a highly technical and scientific form of curative treatment.

We cannot help feeling that it would be well if medical men would satisfy themselves, before employing masseurs or masseuses, that they possess such documentary qualifications (giving complete proof of training and efficiency) as will stamp them fully qualified to practise a profession that is, alas! far too often abused.

As regards the members of this particular Association, their efficiency has been vouched for by leading members of the medical profession, and for the protection of the public they do not undertake the treatment of patients without the consent and advice of a registered medical practitioner. Under these circumstances it is not surprising that our members are receiving the support of a very large section of the medical profession.—Yours faithfully, F. Chaplin Hall, Secretary.

224-6-8 Great Portland Street,
London, W. 1,
September 17, 1921.

